# The Hospital. Nursing Section

**Published:** 1902-09-27

**Authors:** 


### The Hospital.

### Nursing Section.

Contributions for this Section of "The Hospital" should be addressed to the Editor, "The Hospital" Nursing Section, 28 & 29 Southampton Street, Strand, London, W.C.

No. 835.—Vol. XXXII. *SATURDAY, SEPTEMBER* 27, 1902.

### Notes on News from the Nursing World.

### OUR CHRISTMAS DISTRIBUTION.

As usual in September we remind our readers of our Christmas distribution of useful articles of clothing for the patients in hospitals and infirmaries. Each year the number of articles forwarded to us has continued to increase, and this year, therefore, we propose to make a new departure which we think will be generally appreciated. Before the parcels are sent to the hospitals we shall, if it can possibly be arranged, give an object lesson of what can be done by kind hearts and capable hands in the matter, and exhibit the contributions for a couple of days. Further particulars will be announced later on, but we feel sure that in the circumstances our good friends will redouble their efforts to render help in such an admirable cause, and to make the exhibition as interesting as possible. No names of contributors will be attached to the articles unless expressly desired.

### COMING CHANGES IN MILITARY NURSING.

Among the changes that should be made in military nursing is one which "A Reserve Sister," writing to us on the subject, advocates. The sister of the ward should be, in fact, the mistress of the ward, and in that capacity she should be entirely responsible for its cleanliness and order. Under existing conditions there is a divided authority, and if a sister tells an orderly that things are not as tidy as they ought to be, it is possible for him to retort that the ward master is satisfied, and ignore her reproof. We agree with our correspondent that until this state of affairs is done away with there is sure to be friction; and if, as she expects, the question of the cleanliness of the wards will still rest in the hands of men under the new system, then all we can say is that the new system will excite adverse criticism. The secret of the trouble lies in the presence in the wards of a number of men who are not really ill. If those who are seriously ill are to have the full benefit of being nursed by skilled women, the cases in the wards must be far more carefully classified than has hitherto been done.

### MARRIAGE OF ANOTHER WAR NURSE.

Miss A. A. C. Higgs, who was married on Thursday afternoon last week to Major R. S. de Winton, only surviving son of the late Major-General Sir Francis de Winton, Comptroller and Treasurer of the Household to the Prince of Wales when he was Duke of York, was for between two and three years engaged as a member of Queen Alexandra's Military Nursing Service in tending the sick and wounded in the Transvaal. It was in South Africa that she met her future husband, who was on special duty with a pom-pom section. A novel departure at the wedding ceremony was that the bride, who was given away by her father, the Rev. E. H. Higgs, vicar of Overthorpe, Banbury, carried a silver-mounted prayer-book, the gift of her parents, instead of the usual bouquet. Among the numerous presents was that of a dozen lace handkerchiefs from the nursing sisters at Wynberg and Capetown.

### MEMORIAL TO NURSES.

The endowed bed in memory of the Misses Kayser is to be called the "Kayser Bed." It has just been placed in the New Somerset Hospital, Capetown. The inscription above the bed is as follows:—"In memory of Nurses Ellen and Minnie Kayser, who died of the plague whilst nursing at Uitvlugt, April, 1901. This bed is endowed by public subscription as a memorial of two heroic women."

### THE LATE SISTER CRUIKSHANK.

Great sorrow was felt in the Irene Camp, Transvaal, at the sudden death, from pneumonia, of Sister Cruikshank. The deceased lady, writes a South African correspondent, who for two years was attached to the Yeomanry Hospital at Deelfontein and Pretoria, was much loved by all who knew her, and was most successful in her work in the Irene Camp. She was buried in Pretoria, with full military honours, on July 5th.

### NURSES ON AN EMIGRANT SHIP.

It has been announced in some of the papers that "the transport *Plassy* left Southampton for the Cape on Saturday, carrying a large number of emigrants to South Africa. These consisted principally of bricklayers, masons, painters, carpenters, teachers, governesses, hospital nurses, home helps, and domestic servants." Upon inquiry at the War Office, our representative ascertained that the Colonial Office had taken over the ship, and the authorities there stated that "no nurses were sent out by the Government." Those whose names were on the sailing orders—and they were very few in number—were private nurses going to, or seeking employment, sent out under the auspices of the societies for assisting emigration to South Africa. If these nurses have obtained the promise of employment from responsible people upon landing, well and good; but if they have simply obtained assisted passages on the strength of possessing a little capital themselves, we fear that their troubles are only just beginning.

### A NURSES' "AT HOME."

The nurses of Marylebone Infirmary had a very successful At Home on Thursday last week. It had been intended to give a Garden Party in honour of the Coronation on July 4th; this however was of course abandoned when the King's illness was announced, and on account of the uncertain weather it was decided to change the character of the entertainment somewhat. The refreshments were therefore set out in the Boardroom, instead of in a marquee on the "Nurses' Ground," the corridor was draped with flags, and made into a kind of lounge with four o'clock tea-tables and brightly coloured rugs from the nurses' rooms, while busts of the King and Queen had a conspicuous place in the scheme of decoration. A band was engaged to play during the afternoon, and a company of Pierrots performed in the probationers' room, where a stage was erected. About two hundred guests were present, and each of the eighty-three nurses on the staff had the privilege of inviting two friends. Among the matrons who responded to the invitation of the matron, Miss Ramsden, were Miss Vincent (late matron of Marylebone Infirmary), Miss Styring (Paddington Infirmary), Miss Moriarty (Isleworth Infirmary), Miss Morgan (Winchmore Hill), Miss Prosser (Chatham Hospital), Miss Webster (Chelsea), and Miss Blake (St. Pancras), who received her training at Marylebone. In addition, many old nurses were present. The guests were received by Dr. Elliott Browne, chairman, and Mr. Smith, member of the Visiting Committee, and a very pleasant time was spent in the Nurses' Garden, which was gay with autumn flowers.

### PAYING PROBATIONERS.

The alleged hardships undergone by the ordinary probationer who aspires to be a hospital nurse have cropped up again in the daily press. But the *Daily News*, which seems to think that paying probationers are allowed to idle away their time while non-paying probationers do all the work, is not, perhaps, aware that in all well-regulated institutions the scrubbing is done by the wardmaids. As to the polishing, many probationers, instead of complaining of it, take a pride in doing it well. The arbitrary class distinctions to which our contemporary refers, but entirely fails to indicate, exist only in imagination.

### A COUNTY COURT CASE.

It is extremely satisfactory to find a County Court judge warmly espousing the cause of a nurse who has been very badly treated by an employer at Instow. The case, which is fully reported in another column, shows once more that there are still people who think that they can pack a nurse off at a moment's notice on the most flimsy pretext, in spite of a definite engagement at a salary of £30 a year, with board, lodging, laundry, and expenses. The fact that Miss Miller lost her purse in the house where her patient and his wife, with herself, were staying, and was compelled, because of the landlady's attitude, to ask for the advice of a policeman respecting a search, according to her statement, was made an excuse for dismissing her and refusing to pay the salary due. Judge Beresford was so impressed with the injustice of the course pursued that he not only gave judgment for the amount claimed by Miss Miller in the County Court, save an item for board, but said that if the opportunity had been afforded him he would have awarded damages. His decision and remarks are very important, and we advise private nurses to keep the record of the trial for reference and guidance in case they should have the misfortune to undergo a similar experience.

### DUBLIN NURSES INSULTED.

We learn from the lady superintendent of one of the Dublin hospitals that a number of nurses who answered an advertisement have been "grossly insulted." The advertisement was as follows: "Wanted, a female nurse for a rheumatic patient. One with a knowledge of massage preferred. Liberal terms will be given. Apply —— Hotel, Dublin." The advertisement appeared in three different Dublin papers on three different occasions, and in each case the name of a different hotel in the city was given. There is nothing on the face of the advertisement which would justify the refusal of the publishers of the papers to insert it, but if the nurses who were insulted had at once given information to the Dublin police proceedings could have been taken.

### A UNIQUE ENTERTAINMENT AT PORTSMOUTH.

The Parish Infirmary at Portsmouth has a supply of flowers most of the year round forwarded by "Uncle Taff" from the members of the League of Love, which also sends toys at Christmas to many institutions besides the infirmary. On Saturday last the chairman of the infirmary committee and the medical superintendent, Dr. Chas. Knott, entertained the members of the League, and of its kind the meeting was unique. It was the first in the neighbourhood, though the community has been working since 1893, and the members had not before had an opportunity of seeing one another and meeting "Uncle Taff." Moreover, they had never seen the infirmary. The company, numbering about eighty members, mostly quite young girls, with a few outside friends invited to meet them, gathered on the lawn by the nurses' home, where the boys' band was stationed. After a few introductions they were allowed to see a ward or two; next they had tea on the grass, and after that a few games; two or three speeches then brought the meeting to a close, and the company dispersed. The members went away more eager to follow up their work and to bring, wherever possible, a little brightness into the lives of the sick and suffering. All in the infirmary, both patients and staff, join in thanking "Uncle Taff" and the League of Love, and in wishing them every success in their good work.

### THE INTRICACIES OF PRIVATE NURSING.

Writing in the monthly paper of the Guild of St. Barnabas for Nurses, Miss C. J. Wood refers to the alleged discontent of the public with trained nurses. She admits that there are black sheep, but insists that they are few in number compared with those who spend themselves for their patients, and whose sympathetic devotion sustains the household in the hour of trial. She continues, "No nurse, however well trained, can understand the intricacies of private nursing. The friends cannot be treated as in the hospital, the patient must be studied, the servants are not just ward-maids to be sent hither and thither at the will of the nurse, and even the doctor comes in unfamiliar guise. Where are the divine gifts of humility and patience that wait and adjust themselves to the needs of the moment? Shyness or nervousness are often responsible for the brusque, unsympathetic manner which gives a bad impression, and may stay with the nurse, although often hiding a warm loving heart, lacking the means of outward expression."

### MEDICAL ATTENDANCE AND INFIRMARY NURSES.

There has been a discussion among the members of the Yarmouth Board of Guardians as to whether the nurses in the workhouse infirmary are entitled to medical attendance in case of illness. Of course, it was not denied that if a nurse contracted a contagious disease in the discharge of her duty the guardians would be under a moral obligation to provide a doctor. But it was urged that they had no legal responsibility in the event of a nurse falling ill. It is, however, clearly the view of the Local Government Board that nurses should not be called upon to pay doctors' fees. While they have refused to allow a direct payment for attendance upon the nurses at Yarmouth, they have consented to an increase of the salary of the medical officer in order that he may attend upon them free of charge. In this way it is obvious that proper provision can be made, but if a board of guardians decline to give the necessary increase of salary to the doctor, either the nurses or the doctor must suffer in pocket.

### CHELMSFORD WORKHOUSE AND INFIRMARY.

The nursing arrangements in the Chelmsford Workhouse Infirmary may not be satisfactory; but if, as we understand, the man who recently died in the night was without warning taken ill in one of the able-bodied wards, we do not think the committee of investigation of the Board of Guardians can be fairly called over the coals for finding that there was "no one to blame in the matter." As we intimated a fortnight ago in reference to a case at Lambeth, there is no reason why a nurse should be asked to enter the dormitories containing able-bodied paupers. In fact, she has a right to object to do so, and the inmates have also a right to object to her visits. There is, of course, always the chance that a person may be suddenly seized with illness, whether in the workhouse or the home, but such emergencies can only be provided for as they arise. When the inmate of the Chelmsford Workhouse asked for assistance, the night nurse on duty appears to have come to him at once and to have done what she could for him. That he expired in a few minutes from syncope is no justification for asserting that there are not enough nurses in the workhouse infirmary, and demanding the intervention of the Local Government Board. It may, however, have been a justification for a complaint that the man was left in the able-bodied ward.

### NURSES FROM SOUTH AFRICA.

The following nurses have arrived in Southampton from South Africa since our last list was issued:— On the *Assaye*, Sisters M. Bell and M. Morrison; on the *Donne Castle*, Sisters H. Anderson and C. S. Carse; on the *Scot*, Sisters A. F. Bedwell, M. J. Martin, and J. Speed; and on the *Sunda*, Sisters A. Knaggs and H. Hogarth.

### COTTAGE NURSES IN CUMBERLAND.

A bazaar has been held at Penrith in aid of the funds of the Cumberland Nursing Association, which has now been established nearly five years. The takings amounted to £1,340, a very substantial result. Starting with a staff of eight, there are at present twenty-seven young women working under the Association, and there are seven more still in training at Plaistow. The term of their training is nine months, and the cost in each case is £52. We cannot help feeling, however, that "the towns, villages, and scattered districts of Cumberland" in which they are engaged, must remain at a disadvantage compared with other counties where fully-trained district nurses are employed.

### SIR ROBERT HARVEY AND NURSING HOMES.

Under the title of "Nursing Homes: A Warning," Sir Robert Harvey, of 1 Palace Gate, W., and Dundridge, Totnes, has published a pamphlet setting out a statement of claim on his part in an action brought by him against Harriett Maria Webb, 88 Marina, St. Leonards, claiming damages, £156 8s. 3d. The claim was made on the ground that Sir Robert's son, a boy of ten years old, who had been received into the defendant's home while he was suffering from a slight attack of German measles, contracted diphtheria, from which he died, owing to the omission and neglect of Miss Webb to use the reasonable care and skill in the management of her business upon which he relied when placing the boy in her charge as a skilled nurse. The defence of Miss Webb was that there were neither omissions nor neglect, and that she used reasonable care and skill in the management of her business to prevent injury to the health of all persons whom she received in her home to lodge, board, and nurse for payment. On August 5th Sir Robert Harvey's solicitors were informed by the solicitor to the defendant that he had issued a summons for an order, "that this action be stayed upon payment by the defendant to the plaintiff of the damages claimed in the statement of claim and the plaintiff's taxed costs of the action." The order was necessarily made, and the case cannot therefore be tried.

### LEVELLING UP AT BURNHAM.

The members of the Nursing Association at Burnham, in Essex, have very wisely decided to provide "a trained and competent nurse," to minister to the wants of the poor. At a special meeting of the Association a resolution to this effect was carried in face of an amendment which, if passed, would have resulted in the Burnham Association being affiliated to the County Cottage Nursing Association.

### SHORT ITEMS.

Mrs. Mather, late lady superintendent of the London County Council Home for Inebriates at Farmfield, Surrey, and formerly matron of the Royal United Hospital, Bath, was married last week at All Souls, Langham Place, to Dr. Williamson.—The Bath Board of Guardians have referred to the Hospital Committee the question of appointing additional nurses. One member of the Board is of opinion that the staff is already excessive.

### Lectures to Nurses on Medical Nursing.

By Eveline A. Cargill, M.D.Brux., L.R.C.P.&S.Ed.

### LECTURE VII.—DISEASES OF THE LUNGS.

Diseases of the air passages are accompanied by cough and dyspnœa. Cough may be of many kinds and degrees, and is generally curative in action, as by coughing we get rid of substances which are obstructing the air passages. As a rule, the more violent a cough is the less serious it is; very hard noisy coughs are caused by irritation of the tonsils and upper part of the throat, and are frequently kept up by digestive disturbances. The cough of bronchitis is an exception to this rule; it is a distinctive cough, large, loud and loose, and it brings up large quantities of frothy mucus. It is not much affected by talking, whereas cough due to irritation of the throat is made worse by talking. There is not much cough in pneumonia, and what there is is sharp and short. Phthisis has a slight hacking "irritating" cough in the early stages. Cough may be relieved by inhaling warm, soothing vapours (steam), by warm drinks (such as warm milk, sipped slowly), or by cough medicines. The latter should never be given except by medical advice, especially to children, as many stock "cough mixtures" contain opium, and opium may check the cough but it may also kill the patient. Children are extremely sensitive to opium, even in very small doses, and require most careful watching when the drug is being administered by the doctor's orders.

Dyspnœa, or breathlessness, denotes insufficient aeration of the blood. It is more severe when the smaller bronchial tubes are blocked, and when severe is accompanied by lividity of ears, nose, lips, fingers, etc., due to the venous state of the blood. When this is the case, inhalation of oxygen gives relief; compressed oxygen is supplied in iron cylinders fitted with a rubber tube and screw; the gas may be allowed to play gently over the patient's mouth and nose for ten or fifteen minutes at a time, and repeated whenever the breathlessness and lividity recur. The oxygen so given supplies to the blood what it cannot easily obtain from the air in the lungs. For breathlessness warm inhalations are also useful, and warm moist air is soothing to the air passages, and is best provided by means of a bronchitis kettle. A sharp attack of dyspnœa may be relieved by a diffusible stimulant such as half a teaspoonful each of sal volatile and spirits of ether in a wineglassful of water. When there is much dyspnœa a patient may find it difficult to breathe lying down, and should be propped up by pillows or bed-rest in a sitting position. The clothing should be warm and light with no bands round chest or waist.

In all diseases of the air-passages the ventilation of the sick-room requires special attention. It is essential that pure, fresh air, containing abundance of oxygen, be supplied to the lungs, which, owing to disease, are seriously hampered in their work, and free ventilation must be ensured day and night. A cross current of air keeps the room fresh and pure; a window (not near the bed) open at the top, with an open fire is a good ventilator, and in summer a lamp burning in the fireplace keeps the air moving without giving out appreciable heat. The patient must be protected from "chill" or "draught" by light curtains or screens, but heavy hangings to bed or window are undesirable. Gas should never be burnt in a room where there is lung disease of any sort. The temperature of the room should be rather higher in throat and chest cases than in other illnesses and should be kept as evenly as possible at 65° day and night; it should not fall below 60°, nor rise above 70°. A bronchitis kettle should not be too near the patient (unless specially ordered), the object being to moisten the air of the room, not to puff steam on to the patient, and care must be taken that the moisture is not overdone and the room converted into a vapour bath.

Bronchitis, or inflammation of the bronchial tubes, may occur at any age, but is most common and most dangerous at the extremes of life, in children and old people. It often accompanies other illness, especially infectious fevers, such as measles, typhoid or scarlet fever, whooping cough, etc. Exposure to cold and damp, insufficient clothing, and underfeeding tend to bring it on, and a variety of occupations involving constant changes of temperature, and probably an insufficient supply of fresh, pure air predisposes to bronchitis as it does to all lung diseases. In bronchitis the bronchial tubes secrete a large quantity of mucus, and it is to the difficulty of getting rid of this mucus which threatens to block the tubes that the distress and cough are due. The cough is at first hard and irritating, later it assumes the character of the well-known "bronchitic cough," with copious expectoration. The cough is wearing, both on account of the muscular exertion it involves, and because it deprives the patient of sleep. But treatment is directed not so much to checking the cough as to getting rid of the mucus in the bronchial tubes, and drugs are prescribed which make this secretion more liquid, and therefore more easily got rid of by cough and expectoration. When a patient does not expectorate freely, an emetic may be necessary to "clear away the phlegm," and is nearly always required in children who seldom can be induced to expectorate.

Bronchitis is relieved by warmth to the chest, and in adults poultices are useful, or a mustard plaster will often cure the tightness of the chest, and can do no harm, except in very old people. But in young children poultices are difficult to manage; they are apt to slip out of position; then also to do good, a poultice must keep hot, and to retain heat it must be of a certain thickness and therefore heavy, and a young child cannot bear weight on its chest. So for a child a spongio-piline fomentation, covered with cotton wool, or a cotton wool jacket, is to be preferred. Still, when poultices are ordered to be applied to a child, if one lung only is affected, the poultice should cover that side only, the other side being left free, or if both lungs are affected, a good plan is to put a poultice over the whole back, and next time over the front of the chest, covering the back with a sheet of cotton wool, and so on, poulticing the front and back alternately. By this plan there is not the same risk of compressing the chest as if the whole were surrounded by a poultice. A child's chest is soft and feeble even in health, and when suffering from lung disease very little pressure may cause "collapse of the lung." For this reason a bandage should never be put all round the chest or abdomen in a child with any lung weakness. A soft woollen shawl is best, loosely pinned on for keeping applications in place. Collapse of the lung is a complication to be dreaded in young children, especially those who are weakly; the signs are pallor of the face with blueish lips, breathlessness, the breathing being quite shallow, with sucking in of the intercostal spaces during inspiration. At any age severe dyspnœa, blueness of lips, etc., and drowsiness are bad signs in bronchitis.

The temperature does not run high, and needs no special treatment. The diet should be light and nutritious, but not necessarily liquid, unless there is much fever, and stimulants are generally required in the latter stages on account of the strain on the heart and exhaustion.

### Private Nursing in North Germany.

BY A CORRESPONDENT.

There are often letters in The Hospital about private nursing. I propose to narrate a few of my experiences in North Germany. To begin with, here private nurses are sent out for 18s. a week, not more, and one institution sends them for 16s. and 17s. per week. I had been taking charge of the medical ward in the military hospital, as the sister had influenza. After four weeks' absence she came back one morning with a message that I was to get ready to go away that afternoon. So at 4.10 I was in the train on my way to my first private case out of Schwerin. It was in March and bitterly cold, snow had been falling nearly all day, so that it was quite 10 and 12 inches deep at 10 p.m. At last I arrived at my destination. It was still snowing hard, the air was bitterly cold, the station was dark, and no one was there to meet me. I had to tell a porter to take my luggage, and follow him, there being no conveyance of any kind. After a good half-hour's walk we reached the house, damp and miserable. How I longed to be back in Schwerin with the sisters in the bright, warm dining-room.

### A Rich Patient.

After knocking at the door a sleepy servant came, telling me all the others had gone to bed, that I was to sleep in my room, but go to my patient at 6 next morning. By this time I was perfectly resigned to fate; even to the sandwiches and a glass of cold milk which she brought me. Next morning, at 5.30, I was called, and at 6 introduced to my patient, who was not amiable either that day or any day. I found she had had a very slight attack of influenza, and the day I was telegraphed for her temperature was a little above normal; but she was immensely rich, very spoilt, and had no children to make her less selfish. For three long weeks I had to stay there, more as a companion than anything else. The only night I slept in a bed was the first night, always afterwards on a sofa in the patient's room, which, of course, one gladly does when the patient is really ill, but when one feels it is hardly necessary it is much more difficult to do so cheerfully. At least four times every night I was called, sometimes to rub the patient's forehead with eau-de-Cologne, or talk, etc. We are supposed to have one hour a day off duty —no more. One day I was ten minutes late from my walk, having lost my way a little, and when I came home was greeted with "You are ten minutes late!" At last, however, to my satisfaction, the time came for leaving.

### A Dear Old Lady.

My next case was a dear old lady of 77, who had had three strokes of paralysis. I grew very fond of her, though, I am sorry to say, she only lived eleven days after I came. Here everyone was so kind and could never do enough for my comfort. It was really touching, and the more so because it was such a contrast to my former experience; though here too I only went to bed once properly and had to be up by day as well. The daughters, it is true, used to sit up at night, but if I had retired to rest I should have been uneasy all the time, knowing no trained nurse was there. Moreover, I knew that they felt safer having someone there, and that they could ill afford two nurses. I do not take credit for so doing; all our nurses would do the same willingly. I often feel ashamed to grumble when I see how devoted, as a whole, the sisters are and how little they think of themselves; what few pleasures they get and how contented they are, generally speaking. At length the sad day of the patient's death came. She was truly loved by all who knew her. Even in the last hour she was conscious, though she was dying from Saturday midday till Tuesday afternoon. She pressed our hands and tried to thank us.

### A Case in the Country.

After that I had to rest; then came the summer holidays, and afterwards I was requested to take the absent sisters' places. Subsequently, I returned to private nursing again, and amongst other cases I was sent to the country, a place two hours' quick drive from the nearest town and one hour's drive from the nearest station. It was very flat country, no water, and miles of field which may be nice for the owner, but they are decidedly far from pretty. The country house was enormous but hideous. A huge brick building, numerous rows of windows, all straight, no green anywhere on the house, and no trees near. My patient was an old lady, living with her husband, son, and daughter-in-law. She had broken her thigh two years back, then had kidney and heart disease, dropsy, and cancer of the uterus. For some reason or other the family was very unpopular indeed, which made matters worse. During that time I did not have one whole night's rest, for my bed was in the patient's room, where I slept, dressed, and was supposed to have my meals. Only then I did strike. They refused to have another nurse, though very well off, and said that the housemaid might sit up and call me if necessary. I remained for five dreadful weeks for the daughter-in-law's sake, then I was obliged to go. Our committee only sends us to people with the understanding that we can leave after four weeks, provided the people have eight days' warning.

### A Hopeless Case.

My next case after my holiday was a woman with puerperal fever and inflammation of the lungs. It was hopeless when I was sent for—at least, almost hopeless. I went three weeks after delivery. The patient was a good sweet woman, and everyone was kind and nice to me. The first day I arrived, a Sunday, the doctor and I bathed the patient, 27° to 21° C., which seemed to do her good. The temperature was lower, and she herself seemed better. Then the husband said: "Ah, sister, if we had only sent sooner for a sister! But never mind, even now she will get better!" But she grew worse; and on the Monday week that silent change came—no one can explain or describe it, I think—the doctor tried not to believe, but on Wednesday he had to own there was no chance of recovery. It was heart-breaking when the husband said: "She will get better? Yes; sister is here; she must get better." We could not then tell him the truth; but at last even he saw the change—the stare, the uneasy movements, the peculiar whims and fancies. She died in the end suddenly; her last words being: "What is this feeling? tell me, is it death? What light is that?"

### German Nurses.

On the whole I must confess that I disliked private nursing very much, and I am glad at last to have my own work in three parishes to look after and plenty to do; but sometimes I cannot help feeling that German nurses are most devoted and very unselfish. They generally become nurses from a real love of nursing, and not as a means of earning a living in the most respectable way. One is always hearing that "Schwestern must never think of themselves and always do their duty; they may be allowed to object to things

PRIVATE NURSING IN NORTH GERMANY—*Continued.*

as long as they keep it to themselves, for no one can prevent thoughts, but must always do what they are told, cheerfully, whether it is taking charge of the wash-house, or a children's school, a cottage hospital, private nursing, no matter what, they must go where they are sent without expressing likes or dislikes, and be cheerful, and if they cannot do that then they must not become sisters." As to receiving presents, it is most strictly forbidden, and we all are very glad of the rule. Patients can always show their gratitude in many little ways without giving a present. Now as to the work in a private house. We do everything for the patient, and if there is a good deal for the servants to do, we also clean the patient's room and light the fires, etc. But servants or not, we always bring and take the patient's food; and personally I prefer seeing to the room myself, except the grate, or rather stove, which is often a little difficult to light. The sisters are very respectfully treated when they are travelling, etc.; in fact, all classes look upon a Schwester as a woman different from the rest, and the poor specially revere her. Unfortunately, there is a very great scarcity of nurses—as few here as there are many in dear old England.

### Sketches from Life in Bombay Hospitals.

### BANNUBAI'S BABY.

Who was Bannubai? Oh, a person of no importance, none whatever! Only a humble little sweeper woman, taken on as an extra hand during the press of the relapsing fever epidemic in one of our large Indian municipal hospitals A.D. 1900. Only a little outcast, but kind old mother Nature had been lavish in her gifts and had endowed the girl with grace of form and beauty of feature as if to recompense her for the misfortune of being born of a despised race.

Her smooth brown limbs were daintily formed and beautifully rounded. Her face, a perfect oval, was set in a soft tangle of curls that strayed waywardly over the low brow, and her large dark eyes were shaded by sweeping lashes, beneath which they danced saucily. Bannubai was quite aware of her good looks, and dressed herself carefully in brightly coloured jackets and saris, which served to enhance her charms. But alas! Bannubai had her weak point! Though a good worker under supervision, she was incorrigibly lazy, and her great object in life was to do as little as she possibly could.

This child, for she was scarcely more, had not been long in the wards before we discovered that she had another child depending on her. Her first baby was about three months old, and every moment that the young mother could steal from her work was spent with the little creature in the verandah. It slept in a rude hammock, just an old sari slung from the ceiling, and spent its days and nights under the charge of a diminutive paternal uncle, a semi-nude urchin of some nine or ten years of age, with big head and thin legs, Dhondoo by name. Dhondoo used to rock the hammock violently when the baby cried, but if that failed to quiet it he would take it on his meagre lap and toss its head up and down as if it were a shuttle-cock till Bannubai rushed to the rescue, boxed his ears, and relieved him of the small burden.

Like Moses of old, the little Krishna was a "goodly child," with a well-shaped head, square shoulders, and wide chest. Usually his full dress consisted of one little garment, which revealed rather than veiled the soft contour of his velvety limbs. How Bannubai loved that baby! What a pleasure and delight she took in his droll movements and grimaces, looking into our face for sympathy, with an expression of astonishment and pleasure that was curiously bewitching, laughing with child-like merriment when he gurgled and squirmed, wonderingly touching his many dimples, those kiss-prints of the angels, freely bestowed upon the babies of every race. She would seize his little feet and cover them with kisses in the sweet abandonment of motherly love, or lightly twist her pretty fingers amongst his shining sable locks. With so delightful an inducement to idleness, we soon found that if any work were to be got out of Bannubai, she must be treated with great diplomacy, and that the surest way of getting her to labour cheerfully was to spend a few moments in admiring Krishna, or his last new bundi (jacket). "What a good boy he is, Bannubai!" we would say with guile. "He hardly cries at all." "Yes, he is a *chângla moulgá*" (good boy), she would reply, holding him closer to her breast. "All night he has slept and not cried; now he is *bûk lug te* (hungry)." "He is sleeping now," we reply, "so put him in his swing and sweep your ward. It is past five o'clock, and the *dêu-pâlî* (day duty) sister Sahib will be here at seven. You must be *jaldi* (quick)." Thus adjured, Bannubai would deliver her babe to the care of his minute *saga wala* (relation), and condescend to begin her work; but if such small courtesies were omitted, and Bannubai were called peremptorily to her duties, she would perform them in the most exasperating manner possible, sprinkling the floor with water, into which she had deliberately forgotten to pour the disinfecting lotion, or throwing the carbolic powder about in such a manner as to send the whole ward into a commotion, and set all the patients coughing and sneezing together.

The baby grew and throve in the hospital atmosphere. The epidemic was over and the wards were light again, so the *hukum* (order) was given that all the temporary hands should be discharged. Bannubai and Krishna came to offer their farewell salaams. Bannubai made her exit with a woe-begone face, but the baby crowed and laughed aloud as his mother lifted his plump little fist to his brow and bade him "*Salaam! Karu.*")

Last year, in the middle of the epidemic, Bannubai was again taken on the staff. We were very busy, and beyond a friendly recognition had no time to notice her; but as the work grew slacker it struck us that some change had come over the woman. Something of her old happy manner was lacking. Suddenly we remembered the little Krishna, and asked for him. She hung her head and answered almost defiantly, "mele" (dead). "Oh Bannubai!" we exclaimed, shocked and sorry, "How?" "Tap" (fever) she returned shortly. "His teeth were coming, and for many days he had fever. Then all one night he lay this way" (turning up her eyes till the whites only were visible, and breathing hard), "and in the early morning he died." We tried to express our sorrow, but she only answered "*Kaya Karu?*" (what can I do) in a hard tone, and began to sweep vigorously.

It did not need much imagination to fill in the details of the pathetic picture. We could see the little, lifeless body, swathed in snowy muslin, carried by its father and a friend to the burning ghaut. Whilst the young mother, left alone with her grief, a grief that would leave its impress on her as long as she lived, rocked her empty arms for a little while, and then returned to her work with an enduring sigh and the inevitable "*Mi Kaya Karu.*"

### Home-made Hospital Preserves—A Lesson in Housewifery.

BY A CORRESPONDENT.

I remember being struck whilst making a round of the New York Hospitals some time since by one most excellent feature of the well-known Mount Sinai Training School. The Americans are always delightfully hospitable to the stranger within their gates, and I was told when touring through the wards that the housekeeper would be much hurt if I neglected to visit her domestic domains on the basement floor. I think that the least domestic among nurses would have found the array of home-made jams and jellies, preserved fruits and vegetables in that wonderful hospital storeroom a source of joy to her womanly soul. And I learned with interest that some of the nurses of this leading Training School had begged to be allowed to assist in the great preserving festival finished just before my visit. After living for several years in various parts of the United States, I am convinced that American women deserve to rank among the best housekeepers in the world. People who have never been in the States or have stayed on the "other side" for a few weeks only, jump to the mistaken conclusion that most American women live in hotels and boarding-houses, and thus shirk all their domestic duties. I can affirm, nevertheless, that from East to West the average American woman is an admirable housekeeper and a competent cook. My first ideals of hospital housekeeping and invalid cookery—though I was trained in London—were obtained in Philadelphia, the knowledge improved in New York, and the climax of sick cookery capped in Chicago. The commissariat and cooking in the average American hospital is "away ahead" of ours, to use a suggestive native expression. Certainly the preserves at Mount Sinai Hospital would have taken first prize at any domestic exhibition. Made "at home," the cost is so small that the patients constantly enjoy delicious marmalades and jams. Merely to look at the peaches and plums preserved whole gave you an appetite.

### An English Jam-making Matron.

In one or two small country hospitals in England I have met with domestically-minded matrons who have "put up" with their own hands during the summer months, when work in the wards is usually somewhat slack, a certain stock of delicious jellies which no money could buy, for the use of the specially sick. A dear old-fashioned matron who was not trained in the modern sense, but was one of the cleverest nurses I ever met, had charge of a little county town hospital. She used to levy contributions of fruit from every garden in the neighbourhood, from the choicest hot-house melons down to the homely black currant. All the year round she was able, from the resources of her shelves, to tempt her sick with luscious strawberry and other jams, the pips and seeds being carefully strained out of all her produce, so as to fit it for hospital use. There was never a slice of mutton, nor a bit of game, offered to a convalescent without a generous helping of her own currant jelly, to tempt a weak appetite. An unstinted portion of home-made jam or marmalade was served at breakfast and tea for every patient who could take it. The only extra cost to the hospita lay in the sugar. Every bit of fruit was begged from county folk, farmers, and citizens. In country districts people are only too glad to give a small tithe of their garden produce to the local hospital. A little personal begging and persuasion is all that is necessary, and the gifts pour in freely. This model matron has now been dead some years, and the shelves of her storeroom, which used to be the pride of her old-fashioned heart, cannot boast one single pot of any home-made preserve. Her apple and quince jellies were a revelation of the possibilities of preserving. All was pure and wholesome. There was no salicylic or boracic acid to poison the fresh sweetness of the fruit, and interfere with the digestion of her sick charges. Her perfect marmalade, even when she had to purchase the sugar and oranges, cost just three halfpence a pound. You could not buy its equal for any fancy price you chose to pay. Very often she was able to persuade the local grocers to let her have the oranges and other ingredients at cost price. She possessed the tact and talent to interest the nurses, and press them into jam-making service. And this was an admirable domestic training for her young probationers.

### Made in Germany.

In many German hospitals the store-rooms are crammed with bottles and jars of sweet pickles, jams, sauces, and preserved vegetables made on the premises. A supply of such economically prepared dainties gives a note of luxury to the ward diet, and adds variety and pleasantness to the otherwise monotonous menu of sick persons. Not only is an enormous economy effected by making these things oneself, but great care is exercised that no ingredient is added which could injure an invalid. A hospital committee could hardly sanction the serving of such delicacies to the patients if they had to be bought from hospital funds. For it is little "extras" of this kind, bought at fancy shop prices, which mount up so alarmingly in the quarterly bills. In many a German hospital a portion of the meat left from the making of the beef-tea is pounded, passed through a sieve, mixed with a small quantity of lightly-cooked fresh pounded meat, spiced and potted. A small helping of such potted meat served at tea-time or breakfast to convalescent and surgical cases makes a good meal at an infinitesimal cost. The ordinary hospital tea and bread-and-butter meal is a poor one to set before a working man anxious to regain strength. A matron with a soul for housekeeping can devise a hundred appetising methods of using up scraps and odds and ends to vary the hospital commissariat and lighten the terrible monotony which marks the daily ward meals.

### The Moral.

The training of the hospital nurse in this country will not deserve to be called complete until it includes a thorough course of sick cookery and some systematic teaching in hospital economics. So far we have not evolved any satisfactory domestic economy system to apply specially to hospitals, and housewifery appears to be becoming a dead instinct in the rising young generation from which the probationer ranks are recruited. It is a constantly recurring question whether the modern training of the nurse includes too much theory and science and too little practical housewifery. I must confess that in my opinion there is no room for doubt as to the answer.

### Presentations.

Bath Trained Nurses' Home.—Miss F. E. Latham, who is retiring from the nursing profession in consequence of her approaching marriage, has been presented with a silver entrée dish and a silver revolving dish from the committee of the Bath Trained Nurses' Home, and a gold bracelet from the nursing staff. She has also been the recipient of many separate presents from individual nurses.

### A Nurse's Action at Barnstaple.

BY OUR OWN CORRESPONDENT.

A case of considerable importance to the nursing profession came before Judge Beresford at Barnstaple last week, when Miss Grace Miller, a certificated nurse of Salisbury, sued Mr. H. S. Edwards, of Umberleigh, for £6 3s. 3d., made up of 17s. 3d. for wages to June 3rd, £2 16s. for board, and £2 10s. month's wages in lieu of notice. Plaintiff was represented by Mr. Pridham Wippell, barrister, instructed by Mr. J. H. L. Brewer, and her case was that as the result of an advertisement which she inserted in The Hospital defendant engaged her by telegram on May 21st at a salary of £30 per year, with board, lodgings, and laundry expenses, and that on the following day she arrived at Sea View, Instow, where defendant and his wife were in lodgings. Miss Miller's duties were to look after Mr. Edwards, who was suffering from heart dropsy, and for the first six or seven days Mrs. Edwards informed her that she gave the greatest satisfaction. Some little time later Mrs. Edwards complained that the nurse was too much in the patient's room; but the absurdity of this, said plaintiff's counsel, was apparent when it was stated that Miss Miller had to wash and dress Mr. Edwards and also to read to him. It, however, seemed to counsel that this was Mrs. Edwards' real reason for subsequently dismissing his client. On June 3rd Miss Miller lost her purse in the house, and when the landlady (Mrs. Clevesdon) was appealed to, that lady, so far from helping her to find the purse, made some very rude remarks. Having no one else whom she could consult, plaintiff went to the local policeman; but told him she did not want him to go to the house, having regard to Mr. Edwards' state of health. Subsequently plaintiff told the servant that if the purse was not found by 9 P.M. she should send for P.C. Whitfield; and Mrs. Clevesdon, after some insulting remarks, herself sent for the constable, who, on approaching the house, was asked by Miss Miller to walk on the grass lawn in order to prevent Mr. Edwards being disturbed. Plaintiff afterwards found the purse, which contained two valuable gold rings, rolled up in some knitting on a chest of drawers in her own room, and Mrs. Edwards later informed her that she would have to leave next day; that she would pay her railway fare home, and would consult her son, a solicitor, as to what else she would have to pay—she supposed it would be a month's wages. On returning from a short walk, however, defendant's wife ordered plaintiff to leave instantly. She turned her out, bag and baggage, too late to catch a train, and Miss Miller had to remain the night at a neighbouring hotel. Writing plaintiff on June 9th, Mrs. Edwards said in sending her her fare she had paid her more than she was obliged to, her solicitor having advised her that under the circumstances she could dismiss her without any payment at all.

For the defendant (for whom Mr. A. F. Seldon appeared) it was stated that Miss Miller accused both Mrs. Clevesdon and her servant of stealing the purse, and that she created a noise, which was calculated to be detrimental to the patient's health. This, it was contended, was sufficient to justify Mrs. Edwards in dismissing her.

The plaintiff flatly denied the allegations, and his Honour said that Mrs. Edwards had no grounds whatever for discharging her. He believed that plaintiff did her duty, and that she was perfectly justified in coming to that Court and setting herself right with the general public. Judgment was for the amount claimed less the item for the board, which he had not the power to allow. He would have given her damages if possible, as he considered it rather a gross case. Costs were granted on the higher scale.

### For Reading to the Sick.

TRUST IN GOD.

The wind that blows can never kill
The tree God plants;
It bloweth east; it bloweth west;
The tender leaves have little rest,
But any wind that blows is best.
The tree God plants
Strikes deeper root, grows higher still,
Spreads wider boughs, for God's good-will
Meets all its wants.

*Anon.*

How oft a gleam of glory sent
Straight through the deepest, darkest night,
Has filled the soul with heavenly light,
With holy peace and sweet content.

*Anon.*

God bids us, then, by past mercies, by present grace, by fears of coming ill, by hopes in His goodness, earnestly, with our whole hearts, seek Him and His righteousness, and all these things, all ye need for soul and body, peace, comfort, joy, the overflowing of His consolations, shall be added over and above to you.—*E. B. Pusey.*

Watch your way then, as a cautious traveller; and don't be gazing at that mountain or river in the distance, and saying, "How shall I ever get over them?" but keep to the present *little inch* that is before you, and accomplish *that* in the little moment that belongs to it. The mountain and the river can only be passed in the same way; and, when you come to them, you will come to the light and strength that belong to them.—*M. A. Kelty.*

Wait patiently, trust humbly, depend only upon, seek solely to a God of Light and Love, of Mercy and Goodness, of Glory and Majesty, ever dwelling in the inmost depth and spirit of your soul. There you have all the secret, hidd n invisible Upholder of all the creation, whose blessed operation will always be found by a humble, faithful, loving, calm, patient introversion of your heart to Him, who has His hidden heaven within you, and which will open itself to you, as soon as your heart is left wholly to His eternal, ever-speaking word, and ever-sanctifying spirit within you. Beware of all eagerness and activity of your own natural spirit and temper. Run not in any hasty ways of your own. Be patient under the sense of your own vanity and weakness; and patiently wait for God to do His own work, and in His own way.—*W. Law.*

Be content to be a child, and let the Father proportion out daily to thee what light, what power, what exercises, what straits, what fears, what troubles He sees fit for thee.—*I. Penington.*

What have I yet to do?
Day weareth on;
. . . . .
Long though my task may be,
Cometh the end:
God 'tis that helpeth me,
His is the work, and He
New strength will lend.

*Anon.*

### Appointments.

[No charge is made for announcements under this head, and we are always glad to receive, and publish, appointments. But it is essential that in all cases the school of training should be given.]

BLACKWALL BRANCH ASYLUM.—Miss Bessie Ellen Jellett has been appointed superintendent nurse. She was trained at the Middlesex Hospital, London, and has since done private nursing.

BOROUGH ISOLATION HOUSE, PLYMOUTH.—Miss Gertrude Champley has been appointed sister. She was trained at the General Infirmary, Leeds, and was afterwards charge nurse at the South-Eastern Fever Hospital, London. She has since been attached to the Glasgow Co-operation of Trained Nurses.

CALEDON BATHS AND SANATORIUM, CAPE COLONY.—Mr. W. A. Creedon has been appointed head masseur. He was trained at the National Hospital for the Paralysed, Queen Square, London, and has since served as head masseur in the Imperial Yeomanry Hospitals at Deelfontein and Maitland. He has lately been head masseur and instructor in massage to the Temperance Male Nurses' Co-operation, Limited, 15 Great Marylebone Street, London.

CANTERBURY WORKHOUSE INFIRMARY.—Miss Sophy Anne Perrott Field has been appointed superintendent nurse. She was trained at Greenwich Union Workhouse Infirmary, where she has since been staff nurse.

CONSUMPTION SANATORIUM, HESWALL.—Miss Maude M. Bateson has been appointed matron. She was trained at West Derby Union Infirmary, Everton, Liverpool, and has since been night superintendent at Paddington Infirmary and ward sister at Wimbledon Isolation Hospital.

CORK STREET FEVER HOSPITAL, DUBLIN.—Miss Dane has been appointed assistant lady superintendent. She was trained at the Western Infirmary, Glasgow, and has since been staff nurse at Longmore Hospital, Edinburgh, staff nurse at Tooting Hospital, and night superintendent at the Royal Victoria Hospital, Belfast.

CRAIGLEITH POORHOUSE HOSPITAL, EDINBURGH.—Miss Bessie Crowe has been appointed head nurse. She was trained at Brownlow Hill Workhouse Infirmary, Liverpool, and the Nurses' Training Institution, Sheffield. She has since been nurse at Craigleith Poorhouse Hospital, Edinburgh, for six and a half years, and at Dundee Poorhouse Hospital for one year.

DERBY ROYAL INFIRMARY.—Miss Rhoda Graham has been appointed staff nurse. She was trained at the Victoria Hospital for Children, Chelsea; and has since been staff nurse for a year at the Hospital for Incurable Children, Maida Vale, London.

EAST LONDON HOSPITAL FOR CHILDREN.—Miss Violet Watkinson has been appointed theatre sister. She was trained at Birmingham General Infirmary, and has since been sister at the Children's Hospital, Myrtle Street, Liverpool.

FULHAM INFIRMARY.—Miss Annie Bath has been appointed sister. She was trained at University College Hospital, London, where she was afterwards charge nurse. She has since been doing private nursing in connection with St. John's House, Norfolk Street, Strand; pupil and charge at the East-End Mothers' Home, London; district nurse at Poplar, and night nurse at the Acland Nursing Home, Oxford.

GORLESTON COTTAGE HOSPITAL.—Miss Kate Catchpole has been appointed staff nurse. She was trained at St. Mary's Hospital, Paddington, and the Maternity Hospital, Endell Street, London. She has since been district nurse at Greenwich for three years.

GROVE FEVER HOSPITAL, TOOTING.—Miss Daisy Gertrude Bruce has been appointed charge nurse. She was trained at the General Hospital, Wolverhampton, and has since been sister at the Royal Hospital, Boscombe.

LAMBETH INFIRMARY.—Miss Rosa Green has been appointed superintendent of night nurses. She was trained at the South Staffordshire Infirmary, Stoke-on-Trent; and has since been charge nurse at Montgomeryshire Infirmary, superintendent nurse at Nantwich Workhouse Infirmary, and sister of the male surgical ward at the South Staffordshire Infirmary.

LONDON COUNTY COUNCIL HOME, FARMFIELD, HALLWOOD, HORLEY.—Miss Clara L. Cater has been appointed lady superintendent. She was trained at Southwark Infirmary, East Dulwich, and has since been charge nurse at Tooting Hospital, charge nurse at Brentwood Schools, sister on the private staff of the Hollond Institute, Paris, and assistant superintendent at Farmfield.

NEWPORT AND MONMOUTHSHIRE HOSPITAL.—Miss Madge Evans has been appointed matron. She was trained at the Newport Hospital, and has since been sister at Swansea Hospital, and acting matron at the Cottage Hospital, Whitchurch, Shropshire.

NOTTINGHAM CHILDREN'S HOSPITAL.—Miss Courtenay has been appointed staff nurse. She was trained at the Ulster Hospital, Belfast.

NORTH-WESTERN HOSPITAL, LONDON.—Miss Carolyne Moor has been appointed charge nurse. She was trained at the London Homœopathic Hospital, where she was subsequently probationer, staff and private nurse.

NUNEATON GENERAL HOSPITAL.—Miss M. Cecil Lewis has been appointed matron. She was trained at the Royal Albert Edward Infirmary, Wigan, where she was afterwards sister. She has since been senior sister at the Infirmary, Southport, and matron of the Lloyd Hospital, Bridlington.

ROYAL VICTORIA HOSPITAL, BELFAST.—Miss Alice Stewart Glegg Bryson has been appointed assistant matron. She was trained at Crumpsall Infirmary, Manchester, where she was afterwards second assistant matron for ten months. She has also done private nursing in Glasgow for two years.

SELKIRK INFECTIOUS DISEASES HOSPITAL.—Miss Ella Fraser has been appointed matron. She was trained at the Eastern Hospital, Homerton, London, where she has since been charge nurse. She has also been charge nurse at the Tooting Hospital, London, and at Knightswood Hospital, Glasgow.

SKIPTON INFECTIOUS HOSPITAL.—Miss Jane Hutchings has been appointed matron. She was trained at the National Hospital for the Paralysed and Epileptic, London, and the Royal Southern Hospital, Liverpool. She has since been attached to the private nursing home at the Royal Southern Hospital, has been charge nurse at Toxteth Park Workhouse Infirmary, Liverpool, and ward sister at the City Hospital, Birmingham.

ST. OLAVE'S WORKHOUSE, LADYWELL.—Miss Mary Ellen McMahon has been appointed head night nurse, and Miss Gertrude Sarah Powell charge nurse. Miss McMahon was trained at the Children's Hospital, Temple Street, Dublin, and has since been assistant nurse at Cork Street Hospital, Dublin, first assistant nurse at the North-Western Fever Hospital, London, nurse-matron at the Foyle Hospital, Londonderry, and first-assistant nurse at the Long Reach Hospital, Dartford. Miss Powell was trained at St. Olave's Union Infirmary, Rotherhithe.

Stanley Hospital, Liverpool.—Miss Kate Oyler has been appointed theatre sister. She was trained at Guy's Hospital, London, and has since been sister, for one year, at the Royal Portsmouth Hospital.

Ulster Hospital, Mount Pottinger, Belfast.—Miss Julia Parker has been appointed sister. She was trained at the Manchester Royal Infirmary.

Wallasey Cottage Hospital, Cheshire. — Miss Elizabeth A. Stephens has been appointed matron. She was trained at the Royal Infirmary, Preston, and in midwifery at Bristol Royal Infirmary. She has since been head nurse at the Women's Hospital, Derby, night superintendent and sister-in-charge of the operating theatre at the Cancer Hospital, Brompton, London.

### TRAVEL NOTES AND QUERIES.

Rules in Regard to Correspondence for this Section.—All questioners must use a pseudonym for publication, but the communication must also bear the writer's own name and address as well, which will be regarded as confidential. All such communications to be addressed "Travel Editor, 'Nursing Section of *The Hospital*,' 28 Southampton Street, Strand." No charge will be made for inserting and answering questions in the inquiry column, and all will be answered in rotation as space permits. If an answer by letter is required, a stamped and addressed envelope must be enclosed, together with 2s. 6d., which fee will be devoted to the objects of "The Hospital" Convalescent Fund. Any inquiries reaching the office after Monday cannot be answered in "The Hospital" of the current week.

Brussels and Bruges (Jane).—Bruges can be reached by steamer from Tower Bridge, first return 10s. 6d. By Dover and Ostend, first return £2 14s. 11d., second £1 19s. 1d. Brussels by Tower Bridge and Ostend, first return 16s. 9d., second 14s. 5d. By Harwich and Antwerp, first return £2 8s. 6d., second £1 10s. 11d. You do not tell me what style of hotel you want, therefore I cannot be so helpful as I should like. At Brussels the Hôtel de l'Empereur is very comfortable and moderate; pension from 9½ francs. Hôtel de Saxe about the same—both in the Rue Neuve. Much cheaper, but clean and good, Hôtel de la Cathédrale and Hôtel Royal, this last in the Boulevard du Hainant. Still cheaper, Hôtel du Rhin, 14 Rue de Brabant. At Bruges, Hôtel de Flandre, very good, pension from 8 francs; or, considerably cheaper and very comfortable, Hôtel Panier d'Or, this last in the big market square. Very sorry you wrote too late for last week.

Accommodation in Cornwall (2680).—Many thanks for address, which I have noted. Such help is of great assistance to me and to our numerous correspondents.

Employment at Grindelwald (Laconic).—Do not think of such a thing for a moment. There is no such opening. Grindelwald is purely a tourist resort, and sick folk do not resort there, and certainly no one requiring surgical dressings. Accommodation of a very quiet kind co ts about 5 francs per day. Catering for yourself would not answer unless you can speak French or German fluently.

The Canary Islands (S. P. S.).—Thanks for your communication. I will remember what you tell me in the event of correspondents going there, but the journey is too long and expensive to make an article on so distant a spot of sufficiently general interest to our readers.

### Everybody's Opinion.

**[Correspondence on all subjects is invited, but we cannot in any way be responsible for the opinions expressed by our correspondents. No communication can be entertained if the name and address of the correspondent are not given as a guarantee of good faith, but not necessarily for publication. All correspondents should write on one side of the paper only.]**

### A HARD CASE.

"Lillian Davidson," of the East London Nursing Society writes from Church House, Buxton Street, E.: May I enlist sympathy on behalf of a poor district nurse who last week had the misfortune to have her pocket picked and lost her purse, containing a month's salary. She is poor and friendless, and that money was all she had in the world. Any little help would be most gratefully received.

### HOW TO RELIEVE SNEEZING.

"Nostrum" writes: Referring to "Notes and Queries," No. 138, I should like to give my experience upon violent sneezing and its remedy. The sneezing I am speaking of comes on without any reason as far as can be discovered. It cannot be attributed to the usual causes, such as cold, draughts, dust, or a strong scent, but comes on suddenly, the sufferer sneezing constantly without stopping. Then there is a moment's cessation, followed by another attack of sneezing. I have seen this continue for hours until the poor sufferer was totally exhausted. The following treatment I have found most of relief. Cause the patient to assume a recumbent position; if able to retire to bed so much the better. See that she is warmly wrapped in a blanket or has a hot-water bottle. Let her try a sudden good smell of "smelling salts" or a little powdered boracic acid taken like a pinch of snuff. A glass of hot milk revives the fainting energies of the sufferer, who is kept warmly in bed, not to prevent cold, but because the process of sneezing is so exhausting.

### THE HOME SISTER'S AUTHORITY.

"A Liverpool Nurse" writes: May I be allowed to say, with all due respect to Nurse Elizabeth, that I trust she will never occupy the post she has so bravely defended. Otherwise, it will indeed be hard lines for the nurses. I expect that Nurse Elizabeth has been so taken up with defending the home sister that she has unfortunately muddled up one letter with another. The reference she makes to sick nurses has nothing whatever to do with me. As the two points I asked for information about, namely, liberty in off-duty time and free choice of dress, have been gained, I thank you for your kindness in publishing my letter, as it has been the means of bringing about a better understanding, and the discussion has been one of interest to all.

"A. P." writes: Having read the letters in your paper *re* sailor hats and dirty boots, may I venture to ask a question? Would Our Lord wish us to stay away from the services of His church or chapel simply on account of a hat? No doubt we all have our little vanities and like to look nice if we can, but there is a wide difference in our circumstances. Some nurses can spend much on dress and amusements, and when their money is gone can write home for more; others out of their small pay have to keep not only themselves but, in part at any rate, an invalid mother or sister as well. The home sister cannot always be the best judge of a nurse's means. As regards boots, I used to find it necessary to keep at least one pair always ready and clean as the time for changing and going out, getting back into uniform again, and putting things away was never long enough. But surely if we are considerate and patient with one another these small matters adjust themselves. We all know that a nurse needs patience above all things. In this month's "Church Monthly" the Bishop of Chichester writes about Sunday-school work, says: "We must have patience! patience with others, patience with ourselves, patience with God" (*i.e.* Faith). "Patience is not apathy or callousness; it is a lively quick-souled thing. It can endure pain calmly, suffer fools gladly, and harder still, can bear successes without undue elation." Do not these wise words apply quite as truly to nursing as to Sunday-school teaching; and would not all friction between the home sister and the nurses disappear if on both sides a little more patience were shown? We know well enough what it is to be tired and depressed and sometimes worried at having failed to reach our ideal; but we shall not improve matters by being impatient either with our superiors or with our subordinates. [This correspondence must now cease.—Editor, The Hospital.]

### DISTINGUISHED SERVICES.

"A Small-pox Nurse" writes: As you stated, under the heading of "Distinguished Services," there is very little risk of a nurse catching small-pox, assuming that she has been properly vaccinated. But a nurse has to undertake the following risks if she wishes to nurse small-pox: She may get eight teeth knocked down her throat by a bang from a delirious patient, or a blow on the head from the same source, which incapacitates her for an indefinite period. Or a patient may be subject to a fit of choking whilst the nurse is feeding him or bathing his eyes, and thereby get his saliva into her own eyes. This may cause her months of suffering, and it is doubtful whether she may regain her sight. It is her duty to spend 12 consecutive hours in a poisoned atmosphere owing to the odour which comes from the confluent and hæmorrhagic cases. When a nurse first informs her relatives and friends that she intends to nurse small-pox many try to dissuade her. If she still insists on going they may add, as they did in my case, "For God's sake, don't come near us!" And when she is there they may refuse to receive letters or have anything to do with her. There are many nurses who have not seen a friendly face since they entered Long Reach Hospital, and, although they have good off-duty times, have nowhere to spend them. Perhaps it is not generally known that the authorities are most particular that no nurse leaves their gates without first having a bath and their hair washed with strong carbolic soap. Moreover, the minutest article of their dress which they are about to put on passes through the disinfectant machine. This process is gone through every time the nurse signifies her intention of going out, and there is a special female attendant to see that all these orders are carried out. Yet, with all this, people are afraid of us, and do not say kind things should we enter a railway waiting-room during a downpour of rain, and it leaks out that we had come from Dartford. When nurses leave the hospital, they in most cases find it difficult to get lodgings, especially those who have no homes of their own. And when they do get them, the prices are exorbitant according to their salary. So we Englishwomen do not grudge Miss Kemner the reward she deserves. She has had to suffer isolation, if nothing else.

### SURGICAL DRESSINGS.

"J. B." writes: When I was in America last year I visited a factory for surgical dressings at New Brunswick, and was most interested in the numerous processes by which the raw cotton was changed into absorbent wool and gauze, and the absolute cleanliness of everything. After going up and down endless stairs, and looking at all sorts of wonderful machinery, we came to the laboratory, which consists of three individual buildings with four large rooms, one for absorbent wool and the others for gauze. The process of cleansing and purifying is carried on in large vats, those for cotton wool having a capacity of 5,000 lb. each, and those for gauze 52,000 yards each. In all the processes neither the wool nor the gauze is handled. In the first place, the mechanically cleaned cotton enters the laboratory by a current of air, and, after it is made absorbent, endless belts carry it to the dryers. The gauze enters the laboratory in the form of an endless rope, and is drawn over rollers from one operation to another. As the gauze leaves the looms it is pulled down into this laboratory, where it passes from solution to solution, is rubbed, flooded with water, douched, irrigated, and finally wrung, stretched, dried, and rolled ready for medication, sterilisation and packing. It is then taken by machines which measure, fold, and pack it in glass jars and packets ready for sterilising. The whole factory is remarkable for its cleanliness; the floors and walls are constantly washed, dirt and litter are prevented from gathering by forced draughts of purified air. We were only allowed to look into the packing room through glass doors, as no one is allowed in without sterilised clothes. I understood from the manager that the packers we saw in the packing room were trained nurses, and before going into the room they washed their faces, their nails were manicured, and hands and arms scrubbed and disinfected, their hair was completely covered by large sterilised caps. After the wool has been packed and put into cases it goes into a steriliser, where it is finally sterilised by formaldehyde gas. In being as far as possible consistently aseptic, the Americans, on the whole, I should think, were pretty hard to beat, and if the nurses who open the packets of gauze and wool are as careful as those who pack them, there ought to be very little danger of sepsis.

### MILITARY NURSING.

"A Reserve Sister" writes: A few words about military nursing may be acceptable at the present moment when so many are thinking of taking up this branch of work. Many changes are to be made, we learn, during the next few months in the military nursing world, but the question is, Will these be the changes most likely to do good? Those who are now engaged in military nursing, and have been for years, think it the greatest mistake possible to suppose that any nurse having already had a three years' training will think it worth her while to enter a military hospital as a nurse with very little prospect of being promoted to a sister's post, even after having served three years more and it is the ambition of most nurses, who are worth anything to at least become a sister by the time she has been nursing six years. At the same time there is little doubt that the present system is wrong, and requires thoroughly reorganising. The cleanliness and order of a ward are seen to by the wardmaster and those under him in the present state of things, instead of being, as in civil life, under the entire control of the sister of the ward. The consequence of this is that if a sister thinks the wards she is working in not as she would like them to be either as regards cleanliness or tidiness and takes on herself the task of reprimanding the orderly, it is quite on the cards that he will tell her that as the doctor and wardmaster have not complained she has nothing to do with the matter. Until this state of affairs is done away with there is sure to be friction. But as the orderlies who will be retained to do the cleaning of the wards under the new system will be working under the wardmaster and the nurse under the sister the question of cleanliness still will rest in the hands of men. Surely this is a woman's work more than a man's! Again, is the nurse to sit in the wards when not busy? "Tommy" will not take kindly to nurses sitting in the wards, and as far as one can judge it will be a very unpleasant experience for those who are the first to set the example. In nine wards out of ten, apart from enteric wards, there are probably not more than two patients in each ward in a military hospital who do not get up part of the day. One rather wonders, too, what will happen when Tommy wants to take his afternoon nap, which he religiously enjoys after having his dinner, getting into bed very often with his trousers on, and afterwards going out for a walk till tea-time. To those who have done military nursing for years the idea of nurses sitting in the wards seems almost an impossibility, and yet one cannot help seeing that if it is workable—which only a trial can prove—the comfort of those who are really ill will be greatly added to by the constant attendance of a thoroughly trained nurse rather than by the attentions of a willing, but very often clumsy and untrained, orderly recruit. The nurses chosen for this work should be women of dignity, some culture, and plenty of tact, otherwise there will be nothing but trouble in store for the authorities, and Tommy will look upon the nurse as his social equal, and therefore a person to whom he can and will offer his attentions if not kept in his place. If the selection is carefully made, and not in the promiscuous way that the reserve nurses were chosen for South Africa, the scheme should be a success; but if not, what has hitherto been looked upon as a select service will deteriorate, and that very rapidly.

### Echoes from the Outside World.

### The Royal Progress through London.

It is now officially announced that the royal progress through the streets of London, which was appointed for June, the day following that fixed for the Coronation of the King and Queen, will take place on Saturday, October 25th, not October 18th as originally and unofficially stated. The exact route to be traversed will be published hereafter. The Lord Mayor of London has received an intimation from Lord Knollys that their Majesties will lunch with the Corporation of the City at Guildhall at one o'clock on the same day. On the following morning, October 26th, the King and Queen will attend a Coronation Thanksgiving Service at St. Paul's Cathedral. It is expected that on both occasions their Majesties will be accompanied by every member of the Royal Family then in England. The King has expressed his regret that he is unable to undertake to pay a visit to the East End as well as to South London next month.

### The Queen of the Belgians.

It was known that the Queen of the Belgians had been suffering from asthma and heart weakness of late, and that no improvement could be reasonably hoped for, but her death, on Friday evening at Spa, came as a shock to her own people as well as to others. She was seated at the table partaking of a light meal, when she was seized with sudden cardiac trouble, and although the doctor in attendance upon Her Majesty was summoned at once, the Queen died of syncope before he could reach her. Marie Henriette Anne was the youngest daughter of the Archduke Joseph, Palatine of Hungary, and was aunt to the Queen-Mother of Spain and the Duchess of Orleans, and great-niece of Marie Antoinette. On the eve of her 17th birthday she was married to the Duke of Brabant, heir to the throne of Belgium. The Belgians were most enthusiastic over their future Queen, and were so captivated by her beauty that they christened her "The Rose of Brabant." As a young married woman she was frequently in England, and seemed much attached to her husband's cousins, Queen Victoria and the Prince Consort. Three of her children were born before her husband came to the throne in 1865, and one afterwards. But sorrow after sorrow came to her through those to whom she was most devotedly attached. First of all her kinsman, the Emperor Maximilian of Mexico, was assassinated, and she felt the blow keenly. For 30 years she charitably tended and cared for her bereaved sister-in-law, Maximilian's widow. Then at the early age of 10 her only son, whom she adored, was taken from her. The little boy's tutor, Mr. Charles Murray, a Scotchman, was retained at the Belgian Court long after his charge's death, and, indeed, accompanied the King of the Belgians to London on the occasion of Queen Victoria's first Jubilee. Princess Stephanie, her second daughter, married the ill-fated Crown Prince Rudolph of Austria, whose death, under most distressing conditions, will be remembered. The marriage of another daughter was not a happy one. The Queen of the Belgians was extremely fond of dogs and horses. It was said that she could break in any colt, and she had a large riding school at Laeken, her favourite castle, which was burnt to the ground in 1890, numerous priceless treasures being destroyed. The Queen was also a clever musician, a modest authoress, and of a most charitable disposition. The Pope bestowed upon her the Golden Rose in token of her piety. The interment, according to the wish of the late Queen, was of the simplest character. The body was taken from Spa straight to Laeken Church, not first to the Palace in Brussels, and the funeral took place at four o'clock on Monday. It was strictly private, and no foreign representatives whatever were present. King Edward has commanded that the English Court shall wear mourning for three weeks for the Belgian Queen.

### Operation on President Roosevelt.

The public engagements of the President of the United States were suddenly cancelled on Tuesday, in consequence of it being found necessary to perform an operation on Mr. Roosevelt at the St. Vincent Hospital, Indianapolis. It appears that one of the bruises on the left leg, between the knee and the ankle, received by the President on the occasion of the recent trolly accident, developed into a small abscess. This, in the opinion of the doctor, needed an operation, which accordingly took place on Tuesday afternoon. The President was not placed under anæsthetics, and remained in good spirits. After the operation he was placed in bed, and he is reported to be doing excellently. The doctors state "that the case is in no way serious, and that there is no danger whatever." But Mr. Roosevelt will require entire rest for at least 10 days.

### Permits to South Africa.

The announcement that martial law has been abolished in Cape Colony has caused many emigrants, anxious to proceed to South Africa, to jump to the conclusion that no permits are now required. This is true as far as Cape Colony is concerned, where passengers can land at any of the ports. But a British subject wishing to go to Natal, the Transvaal, or Orange River Colony, cannot do so unless he—or she—can produce a certificate signed by a justice of the peace, clergyman, banker, or officer in His Majesty's forces to the effect that he has at least £100, is in a position to maintain himself upon arrival, and is not "deported or sent out of the country as indigent." If the authorities at the Permit Office in Victoria Street are satisfied with the certificate, the permit is issued, personally, to the applicant. This permit, if for the Transvaal or the Orange River Colony, will require to be endorsed by the representative of these colonies at the port of disembarkation. A permit to a husband includes his wife, but no children over sixteen.

### Arctic Explorations.

Captain Sverdup has returned in safety to Norway after four years spent in the Arctic regions on board the *Fram*, the boat which was made memorable by Dr. Nansen's voyage and explorations. He left Norway in June 1898 with the object of exploring the channels between Greenland and the American continent; of traversing the long stretch of unknown coast on the north and north-east of Greenland; and, if practicable, pushing towards the Pole. Commander Peary entered the same field at the same time, and achieved a considerable measure of success; but Captain Sverdup in particular has done great things. The party consisted of 16 men, six of whom were men of scientific training. Geology, botany, zoology, astronomy, magnetism, and meteorology were all represented. The surgeon unfortunately died very early in the expedition, consequently when one of the men dislocated his shoulder while out hunting, having been blown over in a storm, Captain Sverdup had to turn doctor. Once the *Fram* was very nearly destroyed by fire; another time the men were aroused at night by the awful howling of the dogs, and rushing on board they found 12 wolves trying to carry off one of the dogs. Being muzzled the poor beasts could not defend themselves. Several of the explorers had exciting encounters with bears, and once when a depôt had been formed, the bears broke in when the depôt was left and ate up all the dogs' food. Coal beds were found, fiords 70 miles long were sailed through, but no human beings were encountered anywhere.

### Notes and Queries.

**The Editor is always willing to answer in this column, without any fee, all reasonable questions, as soon as possible.**

**But the following rules must be carefully observed:—**

1. **Every communication must be accompanied by the name and address of the writer.**
2. **The question must always bear upon nursing, directly or indirectly.**

**If an answer is required by letter a fee of half-a-crown must be enclosed with the note containing the inquiry, and we cannot undertake to forward letters addressed to correspondents making inquiries. It is therefore requested that our readers will not enclose either a stamp or a stamped envelope.**

*Bleeding after Death.*

(162) Can anyone tell me the cause of the nose bleeding two days after death? The patient was a young man of 22 who died of enteric fever.—*E. B.*

Many more details would have to be supplied before this question could be satisfactorily answered.

*Rötheln.*

(163) Can you tell me of what nature is the sickness called "Rötheln"?

Rötheln is a synonym for German measles.

*Address.*

(164) Would you kindly tell me how I may find out the address of a lady friend, Mrs. Evelyn L. Brougham, who has become a probationer in some hospital in Great Britain? I have not the slightest idea where she is, but I thought that there might be some centre of nursing life through which I might trace her whereabouts. —*Rev. W. B.*

If the lady will send her address we shall be happy to forward it.

*Books.*

(165) *Francesca* would be greatly obliged if the Editor would kindly tell her what books are used by the probationers in the London Hospital.

We understand that the books chiefly used by the nurses at the London Hospital are "Lectures on General Nursing," by Miss Lückes, and "Dr. Hadley's Book on Nursing." In addition, the nurses have access to a large library of medical works.

Will you kindly tell me of any books on paralysis, and where they can be obtained?—*C. P.*

We have not space to catalogue the infinite number of books on "paralysis," a condition of which there are many varieties. Refer to any medical text-book.

*Rhinorrhœa.*

(166) Would you kindly tell me if there is such a word as rhinorrhœa, and what it means?—*Jeanne D'Arc.*

It means a running at the nose, or as the dictionary has it, "The free discharge of a thin nasal mucus."

*Glossary.*

(167) Can you give me information regarding any small glossary of B.P. preparations and ordinary everyday medicines, with the Latin and also the English names alongside?—*R. S. D.*

"A Synopsis of the British Pharmacopœia," by Wippell Gadd (Baillière, Tindall and Cox), is a small book; a larger book is "Squire's Companion."

*Corrosive Sublimate Glycerine.*

(168) Will you kindly tell me how to prepare corrosive sublimate glycerine?—*A. D.*

This should be prepared by a properly qualified chemist.

*G.O.K.*

(169) Will you please tell me what G.O.K. means? Also what is the treatment for the same, if there is a cure, and if the case is a long one.—*J. A.*

This is an admirable example of the folly—or the wisdom as the case may be—of making use of abbreviations. Folly, if the writer wished to make himself understood, but more likely wisdom, if he merely wished to prevent the meaning of his heiroglyphics being understood by his nurse or patient. Obviously these initials mean to the writer exactly what he intended them to mean, to others they mean nothing.

*Training.*

(170) I desire very much to become a hospital nurse; am fully aware that the work is hard, but I am strong and healthy, and not afraid of work of any description. I shall be 19 years old in October. Do you think I could get into a London hospital, Guy's or Middlesex preferably. It is essential that I receive some salary. I do not mind how small, so that I can keep myself quite independently of my father?—*R. R.*

You cannot enter any of the principal London hospitals until you are upwards of 20. The age of admission at Guy's is 23; and at the London and Middlesex 25. At most of the London workhouse infirmaries it is 21, and the salary paid the first year is £10.

*"Don'ts."*

(171) Will you kindly give me the name of the publisher of the "Nurses' Never" and the "Nurses' Don't," I have tried to get them and failed.—*C. B. F.*

The Central Stationery and Printing Company, Liverpool.

*Fruitarian Treatment.*

(172) Will you enlighten those who, like myself, are ignorant respecting the treatment in force at a hospital which is worked on "Fruitarian" lines?—*A. J. B.*

Fruitarian treatment means a diet of nuts and fruit.

*Abroad.*

(173) You mentioned in THE HOSPITAL that a Nurses' Co-operation had been started in Johannesburg, and gave some particulars. Will you kindly tell me from whom I could get reliable information about it, as I know no one in the Colony. I should much prefer taking charge of a patient going out to South Africa. Could you advise me in what paper, other than THE HOSPITAL, to advertise for one?—*Nurse M.*

The Superintendent Nurse, the Co-operative Residential Club, Johannesburg, will be able to give you accurate information on the subject. Any of the well-known medical journals, or even the morning papers, would make your wish known. Then there is now the South African Expansion Committee of the British Women's Emigration Association, Imperial Institute, S.W., the Secretary of which arranges to send suitable emigrants to South Africa by the "indulgence" passages. The cost of an "indulgence" passage is £5 11s., instead of the usual £26 10s., and the emigrants are under the care of a matron both for the voyage and until they obtain suitable employment.

Will you kindly tell me if there are any establishments for English trained nurses in any of the German watering-places? Also can you give me the name of the English Nursing Home at Mentone?—*T. K.*

We do not know of any institutions for the employment of English nurses in Germany. Do you mean the Nice Nursing Institute? The address is Villa Pilate, Avenue Desambrois, Nice.

Will you kindly tell me the name of the Anglo-American Nursing Home in Rome? Is it open all the year, and has it any connection with anyone in London whom one could see or write to?—*Sister Isabella.*

The address is 265 Via Nomentana, Rome. Write for information to the Hon. Secretary, 35A Via Aurora, Rome, and enclose stamps for reply.

To whom should I apply for particulars of nursing in the Riviera? Is there not an institution which sends nurses out for the season?—*M. I. N. H.*, *A. B. W.*, *M. D. P.*, and *A. E. T.*

See reply to *T. K.* The Nice Nursing Institute was known formerly as the Hollond Institution, and used to send nurses out as stated.

Will you kindly inform me where to inquire for information about nursing in Nice for the winter season?—*M. S. J.*

See reply to *T. K.* and *M. D. P.*

Will you kindly tell me how a nurse could go to South Africa to take up private work? I have heard that there is some lady sending nurses out for this purpose, but I do not know who she is Of course, I should like to be sent out and have my passage paid. I would also like to join some institution in order to be sure of employment.—*L. R.*

You have probably heard something about the African Expansion Committee of the British Women's Emigration Association, for particulars of which see our reply to *Nurse M.* You might also write to the Lady Superintendent, the Victoria Nurses' Institute, Capetown, Cape Colony.

**Standard Nursing Manuals.**

"The Nursing Profession: How and Where to Train." 2s. net; post free 2s. 4d.

"A Handbook for Nurses." (Illustrated). 5s

"Nursing: Its Theory and Practice." (New Edition.) 3s. 6d.

"Nursing in Diseases of Throat, Nose, and Ear." 2s. 6d.

"Surgical Ward Work and Nursing." (Revised Edition.) 3s. 6d. net; post free, 3s. 10d.

"Art of Massage." (Second Edition.) 6s.

"Elementary Physiology for Nurses." 2s.

"Elementary Anatomy and Surgery for Nurses." 2s. 6d.

"Practical Handbook of Midwifery." 6s.

"Mental Nursing." 1s.

"Art of Feeding the Invalid." 1s. 6d.

All these are published by the SCIENTIFIC PRESS, Ltd., and may be obtained through any bookseller or direct from the publisher 28 and 29 Southampton Street, London, W.C.

### Travel Notes.

**By Our Travelling Correspondent.**

### CX.—BALLACHULISH AND ITS INTERESTS.

The first evening I spent in this charming place is photographed on my brain by the magnificent sunset effect I then witnessed. I saw many other fine evenings there, but never again such wealth of colour. Morven was a deep ultramarine against a yellow sky, whilst by some subtle effect of refraction, I suppose, the sky on the opposite side was a vivid rose-colour, while the loch itself was the colour of blood from the reflection of the madder sky in its dark waters; the moment was that, when from some trick of the tide, unknown to my unscientific mind, the usually stormy narrows were for a brief time quiet and sullen. This occurs, I believe at what we ignorant folk call the turn of the tide; it was most impressive that brilliant evening, when an almost wierd quiet brooded over the sullen "narrows."

### The Walk up the Loch.

You start for this behind the little hotel and past the Bishop's house. To be Bishop of Argyll and the Isles is no light work, and means considerable athletic exertion and fighting the elements very often. Ballachulish, Glencoe, and the neighbourhood generally is strongly Episcopalian, and there is a charming little church; in the churchyard stands a memorial cross to the late Mr. Machonochie, who, many will remember lost his life in the snow, among the mountains above Ballachulish. He was staying with the bishop at the time, and it was the sagacity of the dogs who were with the unfortunate gentleman that led to the recovery of his body.

The whole distance from North Ballachulish to the Waterfall at Kinlochmore is about 8½ miles; you can cycle all the way, except the climb at the end, and for that you can leave your cycle in a keeper's cottage, where a most kind and friendly woman gave me a sumptuous tea.

### The Appin Murder.

Most people have read "Kidnapped," but I doubt if all know that the incidents therein recorded are founded on facts, and that the terrible death of James Stewart took place at the foot of the blue hill (Creag Gorm), opposite North Ballachulish and close to Cameron's stables. This is the outline of the story, and even to this day it is difficult to unravel all its intricacies, for descendants of the families are still loth to speak freely on the subject. In his preface Mr. Stevenson says: "You will find the tradition of Appin clear in Allan's favour. If you inquire, you may even hear that the descendants of 'the other man' who fired the shot are in the country to this day. But that other man's name, inquire as you please, you shall not hear—for the Highlander values a secret for itself, and for the congenial exercise of keeping it."

In 1752 a certain Campbell of Glenure was employed in collecting rents for the new Government—an occupation, as may be easily understood, sufficiently obnoxious to the proud and loyal Highlanders. Campbell made the unwelcome discovery that the rents were being paid twice over, once to himself for the existing Government, and again to the exiled landowners in France. To stop this he evicted the clansmen from their holdings, and hatred deep and sullen glowed in the hearts of the mountaineers. Tradition says that Campbell was warned, as he crossed from North Ballaculish to the village proper by these words, "I see blood on your plaid." It seems strange that presumably sharing the strong superstitions and faith in second sight characteristic of the Highlanders, he did not pause, but he only laughed and continued his way past the little wood of Lietermore. Here by some unknown hand he was shot dead. A cairn marks the spot. It is quite easily found almost opposite to the Temperance Hotel. At once suspicion fell on one Allan Breck, who was known to have dealings with the exiled Jacobites. It was never proved, however, and undoubtedly local opinion exonerates him. A scapegoat must be found, however, and the unfortunate James Stewart of Acharn was arrested. Nothing was proved against him, except that he had been a friend of Allan Breck's, but the larger part of the jury being Campbells the verdict was dead against Stewart and sentence of death was passed. The condemned man was tried at Inverary by the Duke of Argyll, at that time Lord Justice and General, and he was lodged at Fort William for a few days pending the execution. The prisoner could not realise the fact that an innocent man would really be made to suffer, and refused the offer of the Camerons to effect a rescue on the road to execution.

He was brought to Ballachulish and hanged on the small wooded hillock close in front of the ferry landing stage. When he grasped that mercy was not to be expected he fervently repeated the 35th Psalm, and to this day it is known in the Appin country as "the Psalm of James of the Glen."

The holes where the gallows stood are plainly visible, though 150 years have elapsed, and the peasantry of the district still point out as a proof of the innocence of the sufferer, that no vegetation will grow where the fatal erection stood; this is hardly a veritable fact; but it is certainly true that vegetation there is thin and scanty and seems loth to cover the ill-omened spot.

### Loch Linnhe.

There are endless beautiful walks up and down on both sides of the loch, and if any of your party are poor walkers, the daily steamer leaves you at a desired spot and the return boat in the evening will land you safely at Onich or Ballachulish. Banavie, Fort William, and Oban may be reached in this way. Of this latter place there is so much to be said that I must reserve an account of it for another occasion.

### To Stornoway.

This expedition may be managed by remaining one night at Stornoway, and, to avoid expense, settling to do this the day you leave Ballachulish. Take the morning steamer at Onich or Ballachulish and leave it at Fort William for the train to Mallaig, thence by steamer to Stornoway; the next day return to Oban instead of Ballachulish, and so home. Careful inquiry must be made as to times of starting, for these vary according to circumstances.

Next week I hope to speak of the Pass of Glencoe, before leaving this fascinating neighbourhood.

### To Nurses.

We invite contributions from any of our readers, and shall be glad to pay for "Notes on News from the Nursing World," or for articles describing nursing experiences, or dealing with any nursing question from an original point of view. The minimum payment for contributions is 5s., but we welcome interesting contributions of a column, or a page, in length. It may be added that notices of appointments, entertainments, presentations, and deaths are not paid for, but that we are always glad to receive them. All rejected manuscripts are returned in due course, and all payments for manuscripts used are made as early as possible after the beginning of each quarter.

